# Development of a structural epitope mimic: an idiotypic approach to HCV vaccine design

**DOI:** 10.1038/s41541-020-00269-1

**Published:** 2021-01-08

**Authors:** Vanessa M. Cowton, Ania M. Owsianka, Valeria Fadda, Ana Maria Ortega-Prieto, Sarah J. Cole, Jane A. Potter, Jessica K. Skelton, Nathan Jeffrey, Caterina Di Lorenzo, Marcus Dorner, Garry L. Taylor, Arvind H. Patel

**Affiliations:** 1 MRC-University of Glasgow Centre for Virus Research, Garscube Campus, 464 Bearsden Road, Glasgow, UK; 2 Biomedical Sciences Research Complex, University of St. Andrews, Fife, UK; 3 Section of Virology, Department of Medicine, Imperial College London, London, UK

**Keywords:** Immunology, Microbiology, Diseases

## Abstract

HCV vaccine development is stymied by the high genetic diversity of the virus and the variability of the envelope glycoproteins. One strategy to overcome this is to identify conserved, functionally important regions—such as the epitopes of broadly neutralizing antibodies (bNAbs)—and use these as a basis for structure-based vaccine design. Here, we report an anti-idiotype approach that has generated an antibody that mimics a highly conserved neutralizing epitope on HCV E2. Crucially, a mutagenesis screen was used to identify the antibody, designated B2.1 A, whose binding characteristics to the bNAb AP33 closely resemble those of the original antigen. Protein crystallography confirmed that B2.1 A is a structural mimic of the AP33 epitope. When used as an immunogen B2.1 A induced antibodies that recognized the same epitope and E2 residues as AP33 and most importantly protected against HCV challenge in a mouse model.

## Introduction

There is an urgent need for a vaccine to prevent infection with hepatitis C virus (HCV). Over 70 million people are chronically infected with the virus^1^. Effective drugs are now available to treat chronic infections^2–4^ but we still lack a prophylactic vaccine to prevent primary infection.

A minority of newly-infected individuals (10–40%), clear HCV infection, but the majority develop a chronic infection^5,6^. Successful clearance of virus correlates with a broad, strong T-cell response and rapid induction of neutralizing antibodies during the early phase of infection^7,8^. Importantly, the rate of spontaneous clearance in individuals who have previously cleared the virus increases to about 80% upon reinfection, showing that a protective immune response has been induced, indicating that vaccination is a realistic strategy^9,10^.

The high genetic diversity of the virus—which exists as seven distinct genotypes and over 60 subtypes—makes it difficult to create a single vaccine that will protect against all infections^10,11^. Another obstacle is the presence of immunodominant, hypervariable regions within the envelope glycoproteins^12,13^ which direct the immune response towards non-neutralizing antibody production. The challenge is to create a vaccine that directs the immune response to conserved, functionally important regions, which typically are poorly immunogenic^14,15^.

Much work has been done to identify and characterize antibodies that can neutralize a broad range of HCV isolates by binding to conserved sites of vulnerability on the virus^16,17^. Most broadly-neutralizing antibodies (bNAbs) bind to the E2 glycoprotein and block its interaction with the CD81 receptor^18^. AP33 is one of several bNAbs that bind to a highly conserved region of E2 (residues 412–423)^19,20^. AP33 potently neutralizes all genotypes of HCV in vitro^19,21–23^. It protected mice from HCV infection when passively administered^24^. HCV1, a bNAb which binds to the same epitope, prevented HCV infection in chimpanzees^25^ and reduced allograft rejection after liver transplantation in human trials^26,27^. A molecule able to elicit antibodies similar to AP33/HCV1 would therefore be an attractive vaccine candidate.

The most obvious such molecule would be a peptide corresponding to E2_412–423_. We and others tried this, as soon as E2_412–423_ was identified as the linear epitope of several bNAbs, but immunization with the peptide coupled to KLH never resulted in any antibodies that recognized E2^28^. The failure of the peptide to elicit an anti-E2 response was not surprising, because a peptide is flexible and does not necessarily adopt the same shape as a conformationally constrained section of protein, despite having the same amino acid sequence. Structural studies were then carried out to determine the shape adopted by the peptide when complexed with the bNAbs, with a view to constraining it in a conformation that would elicit the desired antibodies. X-ray crystallography revealed that the peptide, and, by implication, the E2_412–423_ epitope, adopts three distinct conformations: the most potent bNAbs AP33 and HCV1 bind to a β-hairpin structure^29–31^, bNAb 3/11 binds to a double-turn structure while bNAbs HC33.1, HC33.4 and HC33.8 bind to a more open, single-turn structure^32,33^. We attempted to stabilize the β-hairpin conformation recognized by AP33 by making a cyclic peptide immunogen^34^. Immunization with the cyclic peptide did elicit antibodies, but further analysis showed that these antibodies bound a different conformation of the E2 peptide, they had low affinity for E2 and they failed to neutralize HCV.

Here we describe an alternative strategy to create a molecule that will generate AP33-like antibodies. We use AP33 as a template to make a structural mimic of its antigen-binding site (paratope), by exploiting the ability of the immune system to generate antibodies that are specific for the paratope of another antibody. Jernes’ idiotype network theory postulates that the immune system generates a cascade of antibodies that interact with each other^35,36^. Initial exposure to an antigen induces the production of antibodies against it, termed Ab1. The unique paratope is recognized as a set of idiotypic epitopes, or idiotopes, by the immune system. Anti-idiotype (anti-Id) antibodies generated against the Ab1 are termed Ab2. A subset of these, called Ab2ß, complement the paratope of the Ab1 precisely enough to effectively mimic the structure of its epitope on the original antigen. An Ab2ß antibody can be used as a surrogate antigen to elicit Ab3 antibodies, and a subset of these, termed Ab1′, bind to the original antigen^37^. There are several other classes of anti-Id antibodies that do not mimic the antigen, such as Ab2γ, which recognize part of the Ab1 paratope. Distinguishing Ab2ß from Ab2γ can be difficult, because both these classes inhibit the binding of Ab1 to antigen. Numerous studies have demonstrated that Ab2ß anti-Id antibodies can function as vaccines to elicit a protective immune response against infectious pathogens and against tumor-associated antigens^38–41^. Traditional vaccine approaches have failed for highly variable viruses such as HCV and HIV. Both fields are now focusing on the conserved epitopes of bNAbs as leads for vaccine design^42–45^. Here we have used the anti-Id approach to make a molecular structural mimic of the E2_412–423_ epitope on HCV E2. We applied a mutagenic strategy to identify a specific Ab2ß, B2.1 A, which requires the same binding residues for interaction with AP33 as the viral antigen. We demonstrate that immunization with B2.1 A induces Ab1′ antibodies that protect against viral challenge.

## Results

### Production and characterization of Ab2 antibodies

The broadly neutralizing antibody AP33 was selected as an Ab1 as it has proven to be a potent neutralizing antibody that can protect against HCV infection. Analysis of all sequences deposited in the NCBI database using the HCV-GLUE platform^46^ showed that the AP33 epitope is conserved in 93% of HCV E2 sequences (Supplementary Table 1). Only 7% of sequences contain a mutation of one of the contact residues (L413, N415, G418, W420) or an N417S/T mutation that shifts the glycosylation site to N415, any of which disrupt binding^21,29,47^.

**Fig. 1:**
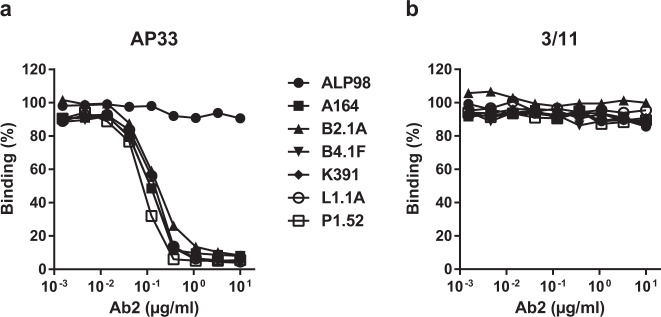
Ab2 antibodies specifically inhibit binding of AP33 to HCV E2. Binding to E2 of (**a**) biotinylated AP33 and (**b**) biotinylated 3/11 in the presence of six Ab2s (A164, B2.1 A, B4.1 F, K391, L1.1 A, P1.52) and an isotype control, ALP98. The values shown are the means of three independent ELISAs. All SDs were within 8% of the mean, and are not shown for the sake of clarity.

Mouse monoclonal antibodies to AP33 were produced and screened by ELISA to identify those that could (i) bind to biotinylated AP33 and (ii) block binding of AP33 to HCV E2 glycoprotein. These anti-idiotype antibodies are rare; in total, we screened approximately 5000 primary hybridoma cell clones from 19 fusion events to obtain 122 that secreted anti-idiotype (Ab2) antibodies. All the Ab2 antibodies were able to inhibit the binding of MAb AP33 to E2 therefore it is likely that they recognize determinants within, or close to, the AP33 antigen-binding site (Fig. 1a). They had no effect on the interaction between E2 and MAb 3/11, which binds to a different conformation of the same E2_412–423_ epitope^21,29,30,32^ (Fig. 1b).

Rather than test all the Ab2s in mice, molecular techniques were used to identify Ab2ß antibodies. Nucleotide sequencing showed that there was no similarity between the Ab2 complementarity determining regions (CDRs) and the E2_412–423_ epitope. Nevertheless, sequencing was useful in that it identified duplicates, which were excluded from further analysis. Ultimately, there were 18 unique anti-Id clones. All the Ab2s only bound to intact AP33 and not AP33 light chain or a hybrid antibody comprising AP33 heavy chain and an irrelevant κ light chain, suggesting that perhaps they were all Ab2ßs^48^.

Using a combined structure- and mutagenesis-based approach, we previously showed that the residues located in the central part of the antigen-binding pocket are essential for binding of AP33 to E2^29^ (Supplementary Fig. 1). A panel of mutant AP33 antibodies, in which each of the residues comprising the antigen-binding pocket was replaced by alanine, proved crucial here for fine characterization of the Ab2s.

The panel was used to determine the “fit” of the Ab2s into the AP33 antigen-binding pocket. We found that AP33 binding of 14 of the Ab2s was severely reduced (>60%) by one or more mutations, indicating that they recognized determinants within the AP33 antigen-binding pocket. In contrast, the binding of some Ab2s was not affected by any mutation, indicating that they recognized determinants outside the antigen-binding pocket. The Ab2s could be ranked according to how closely their binding profile resembled that of E2, and this analysis revealed that only one Ab2, designated B2.1 A, was affected by exactly the same mutations as E2 (Table 1). We thus identified B2.1 A as an Ab2ß that mimics the E2_412–423_ epitope.

**Table 1:** Binding properties of E2 and of Ab2 antibodies to wild type and mutant AP33.

	Relative strength of binding (%)^a^														
	AP33 light chain mutants^b^								AP33 heavy chain mutants^b^						
	Y28	N30	F32	N91	N92	V93	D94	W96	Y33	Y50	Y53	Y58	I95	T97	Y100
**E2**	112	89	**−4**	**7**	60	79	74	**−0.4**	**3**	**−1**	100	**0.7**	**10**	101	**5**
**Ab2**															
B2.1 A	82	73	**21**	**36**	**36**	49	41	**1**	**10**	**4**	68	**5**	**29**	89	**8**
L1.1 A	**5**	88	**3**	85	83	88	86	**1**	**26**	**9**	85	**9**	**26**	84	**13**
P1.T	**20**	88	54	18	48	72	50	**4**	67	**15**	90	**13**	**15**	86	**11**
L1.2 A	76	90	52	31	63	83	88	**1**	**30**	**24**	88	**9**	41	86	54
A16A	93	92	64	57	66	82	70	**7**	**15**	**35**	46	**40**	63	75	**10**
K391	112	74	45	66	82	77	82	**14**	91	**37**	82	41	**35**	98	54
A53M	80	85	55	**21**	86	77	89	**11**	94	88	104	59	54	98	**17**
2K55	87	69	**23**	81	83	84	88	**1**	50	**27**	77	61	60	92	89
2K49	**21**	93	43	94	105	94	97	70	**29**	90	88	91	84	93	**25**
K201	79	90	68	89	115	96	90	**3**	55	**34**	79	67	57	104	82
2K56	89	84	61	88	90	86	94	**1**	57	**39**	74	67	66	90	83
B4.1 F	79	95	49	75	78	100	92	**10**	76	79	94	62	74	97	57
A5	90	98	53	98	105	98	100	**4**	81	54	89	71	74	98	88
2K19	96	88	75	90	94	88	97	**2**	72	54	79	73	75	96	88
A1.5	82	98	94	94	100	96	98	55	93	86	69	85	65	96	78
A164	60	100	66	83	106	98	106	70	73	68	65	93	72	95	59
2K160	78	100	82	86	82	90	104	96	90	91	80	94	91	97	75
P1.52	96	103	79	95	105	106	107	64	84	79	102	71	89	94	80

^a^Binding of E2 and Ab2s to AP33 mutants in ELISA, as a percentage of binding to WT AP33. Each value is the mean of three replicate assays. Alanine substitutions that reduced binding by at least 60% are in bold typeface.

^b^Mutants named according to the identity and position of the amino acid in the WT sequence; e.g., Y28, tyrosine at position 28 of the light chain. Each residue was changed to alanine.

### Molecular basis of antigenic mimicry

To verify that B2.1 A is a genuine mimic of the E2_412–423_ epitope, a Fab fragment of AP33 was co-crystallized in complex with a single-chain variable fragment (scFv) of B2.1 A, and the structure determined to a resolution of 1.8 Å. The structure unambiguously shows the positions of all the amino acid side-chains and of water molecules at the interface between the two antibodies. The asymmetric unit of this Ab1-Ab2 complex was composed of one molecule of AP33 Fab and one molecule of B2.1 A scFv.

**Fig. 2:**
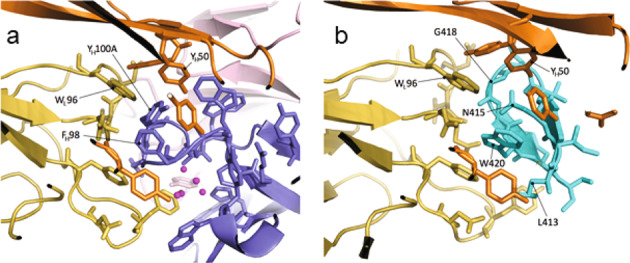
Antigen mimicry by B2.1 A. **a** Ab1–Ab2 complex (AP33 heavy chain: orange, light chain: yellow; B2.1 A heavy chain: blue, light chain: pink) and **b** Ab1–Ag complex (AP33 heavy chain:orange, light chain:yellow; peptide:cyan; pdb accession code 4gag). Water molecules are shown as magenta spheres.

Comparison of this Ab1-Ab2 complex with the Ab1-Ag complex (*ie* AP33 in complex with a peptide corresponding to its E2 epitope (^29^; pdb accession codes 4gag, 4gaj)) shows that B2.1 A docks into the AP33 antigen-binding site (Supplementary Figs. 2 and 3). CDR-H3 of B2.1 A complemented by water molecules mimics the shape and character of the E2 epitope, even though there is no sequence similarity. In general, there is a conservation of the side chain character of the peptide residues critical for the AP33–E2 interaction. B2.1 A mimics the shape of the antigen particularly around the critical E2 residue W420, which is deeply buried in the Ab1-Ag complex. The hydrophobicity of W420 is conferred by several aromatic residues in B2.1 A while W420 itself is mimicked by F_H_98 of B2.1 A in the Ab1-Ab2 complex resulting in the loss of the hydrogen bond (Fig. 2, Supplementary Table 2). The other important E2 residues at the Ab1–Ag interface are G418, N415, and L413. The shape of the antigen around G418 is preserved by the side chain of B2.1 A Y_H_100A, which forms extensive contacts with W_L_96 of AP33. The polar character of E2 residue N415, which is deeply buried in the Ab1–Ag complex, is conferred by the sidechain hydroxyl of Y_H_100A of B2.1 A, which also provides a hydrogen bond to Y_H_50 of AP33 (Supplementary Tables 2 and 3). Interestingly, the interactions of L413 with AP33 are mimicked not by an amino acid residue but by five water molecules in the Ab1–Ab2 complex (Fig. 2). Importantly, this mimicry is also functional with one of the water molecules forming a hydrogen bond to AP33 V_H_Y-100 and bridges to V_H_Y-31 in B2.1 A. Solvent-mediated mimicry has been observed previously at the interface of antigen–antibody complexes helping to overcome imperfections in shape complementarity^49^.

This structural analysis confirms that B2.1 A is an Ab2ß, i.e., an anti-Id antibody that fits into the antigen-binding site of AP33 precisely enough to provide a complementary surface of it, thereby, to effectively mimic the structure of the original antigen.

**Fig. 3:**
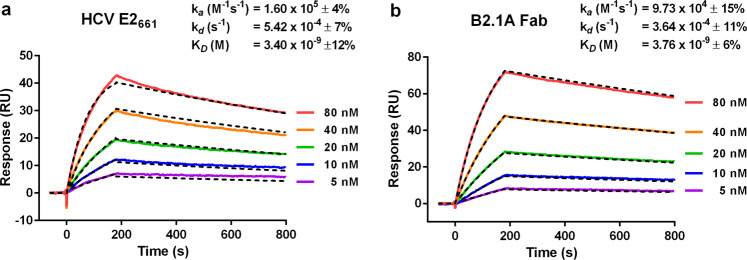
Affinity of HCV E2_661_ and B2.1 A Fab for AP33. SPR sensorgrams showing the binding to and subsequent dissociation from immobilized AP33 of (**a**) monomeric E2_661_ and (**b**) B2.1 A Fab at five concentrations (5 nM to 80 nM). Biacore X100 Evaluation software was used to obtain kinetic affinity constants by fitting a 1:1 binding model (dashed black lines). The kinetic constants shown are the mean and SD of three independent experiments.

Surface Plasmon Resonance (SPR) was used to directly compare the binding affinity for AP33 of soluble E2 that lacks the transmembrane domain (E2_661_) and B2.1 A Fab. The binding kinetics of the two molecules were very similar (Fig. 3). The average association rate constants (k_*a*_) for B2.1 A Fab and E2_661_ were 9.73 and 16.0 × 10^4^ M^−1^ s^−1^, respectively, and their respective dissociation rate constants (k_*d*_) were 3.64 and 5.42 × 10^−4^ s^−1^. Thus B2.1 A Fab had slightly slower rates of association and dissociation than E2_661_, resulting in almost identical equilibrium dissociation constants (K_*D*_) of 3.76 nM for B2.1 A Fab and 3.4 nM for E2_661_. These measurements show that AP33 binds to B2.1 A as strongly as it binds to its authentic antigen, E2.

We did not expect B2.1 A to be a perfect mimic of the E2_412–423_ epitope, and therefore used the structural information, together with protein–protein interaction prediction servers to guide the design of mutations aimed at improving the complementarity of the molecular interface (Supplementary Table 4). However, somewhat disappointingly, all the mutations disrupted the AP33–B2.1 A interaction. Only two mutants V_H_F98W and V_H_N100G retained some binding, albeit weaker than WT, while the remaining mutations ablated the interaction altogether (Supplementary Fig. 4). The reasons for this are not clear, although the protein–protein interaction prediction servers did predict that the mutations based on the structure would disrupt binding. It is possible that our alanine-scanning selection process (Table 1) has selected an Ab2ß that binds AP33 similarly to the AP33:E1E2 interaction thus explaining the analogous binding affinities. Given that B2.1 A already has such a high affinity for AP33, perhaps it is not surprising that we could not increase it by altering any of the amino acid residues within the binding site.

### Immunization with B2.1 A elicits Ab1′ antibodies that recognize HCV E2

**Fig. 4:**
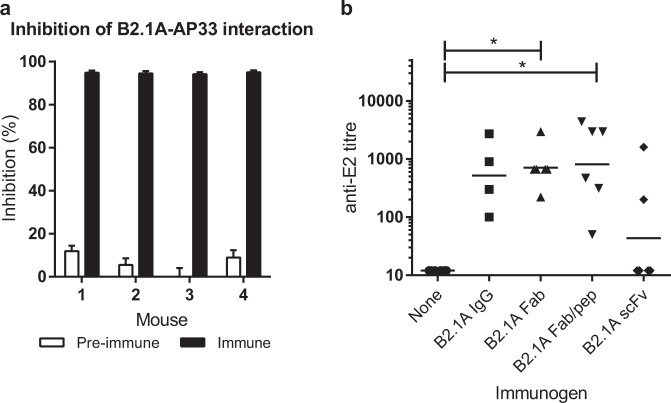
B2.1 A elicits Ab1′ antibodies that recognize HCV E2. **a** Sera of mice immunized with B2.1A-KLH were tested at 1:300 dilution for blocking of biotinylated AP33–B2.1 A interaction. Values shown are the mean and SD of three ELISAs. **b** Balb/c mice were given a primary immunization of B2.1 A IgG, B2.1 A Fab, or B2.1 A scFv followed by five boosters of the same antigen, or a primary immunization of B2.1 A Fab followed by alternate boosters of B2.1 A Fab and E2_411–424_ peptide. The anti-E2 titer of immune sera collected nine days after the last booster was measured by ELISA. Each symbol represents one animal and the horizontal lines indicate the geometric mean of each group; significant differences from the control group are indicated (*p < 0.05).

To test whether B2.1 A could elicit AP33-like antibodies, Balb/c mice were immunized with B2.1 A conjugated to KLH. All the immune sera (a) strongly inhibited binding of AP33 to B2.1 A (Fig. 4a), showing that they contained B2.1A-specific antibodies, and (b) reacted specifically with HCV E2 (Fig. 4b). Given that the constant region of the B2.1 A antibody was unlikely to play any useful antigenic role, we tested whether the Fab fragment or the scFv of B2.1 A, both conjugated to KLH, could elicit the same response as the whole IgG. Immunization with B2.1 A Fab resulted in a higher average anti-E2 titer (700) than immunization with B2.1 A IgG (500), but the difference is not statistically significant due to the small number of animals used and the wide range of titers obtained (Fig. 4b). Immunization with the scFv was less successful, with only two out of six mice producing any E2-specific antibodies. Most of the immune sera also contained a high titer (>5000) of antibodies directed against B2.1 A and KLH, so we attempted to focus the immune response on E2 by giving alternate boosters with a peptide corresponding to E2_411–424_ after a primary immunization with B2.1 A Fab-KLH. This resulted in a slightly higher average anti-E2 titer (800) than that obtained by boosting with B2.1 A Fab-KLH, but the difference between the two boosting regimens is not statistically significant (Fig. 4b). We and others had already established that a peptide corresponding to E2_411–424_ conjugated to KLH did not elicit E2-specific antibodies, so we did not repeat this experiment here. It is significant that we consistently detected E2-specific Ab1′ antibodies in all Balb/c mice immunized with B2.1 A or B2.1 A Fab. We also found that Rosa26-Fluc mice immunized with B2.1 AFab-KLH had a strong immune response, with E2-specific Ab1′ titers above 10000 in most mice (Supplementary Fig. 5).

### Immunization with B2.1A elicits Ab1′ antibodies that bind to the E2_412–423_ epitope

**Fig. 5:**
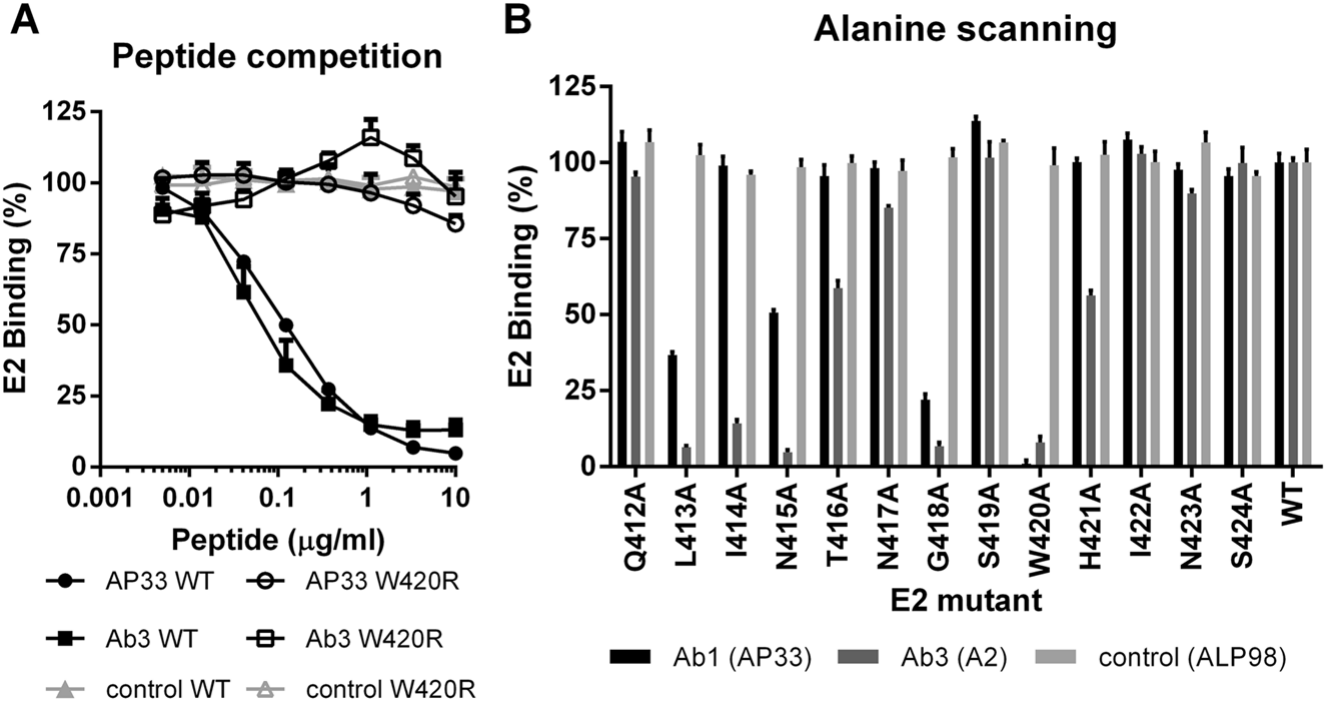
Alanine scanning and peptide competition shows that Ab1′ antibodies bind to the same epitope as AP33. A representative Ab3 serum (1:300 dilution) was (**a**) pre-incubated with E2_412–423_ WT peptide (solid) or with a W420R mutant sequence (open symbols), and then tested by ELISA for binding to E2_661_, (**b**) tested for binding to a panel of E2 mutants, in which each residue in turn from 412 to 424 was replaced by alanine, MAbs AP33, and ALP98 served as positive and negative controls, respectively. Values shown are the mean and SD of three ELISAs. Very similar results were obtained with other Ab3 sera.

Competition ELISA showed that Ab1′-E2 binding was reduced in a concentration-dependent manner by a peptide corresponding to the E2_412–423_ epitope, whereas an identical peptide containing a W420R mutation had no effect (Fig. 5a) indicating that all the E2-specific Ab1′ antibodies in the Ab3 sera recognized W420, an essential contact residue for AP33^21,29^. As expected, MAb ALP98, which binds to a different linear epitope on E2, was not affected by either of the peptides.

Direct ELISA using a panel of E2 mutants, in which each residue in turn across the E2_412–423_ epitope was replaced by alanine, showed that the binding profile of the Ab1′ antibodies in the Ab3 sera was very similar to that of AP33. E2 binding of the Ab1′ antibodies was abrogated by substitution of L413, N415, G418, or W420, the residues critical for AP33 and HCV1 binding. Additionally, Ab1′-E2 binding was reduced by alanine substitution of I414, T416, and H421 (Fig. 5b). As expected, the binding of ALP98 was not affected by any of the substitutions (Fig. 5b).

### Immunization with B2.1 A protects against HCV challenge in vivo

**Fig. 6:**
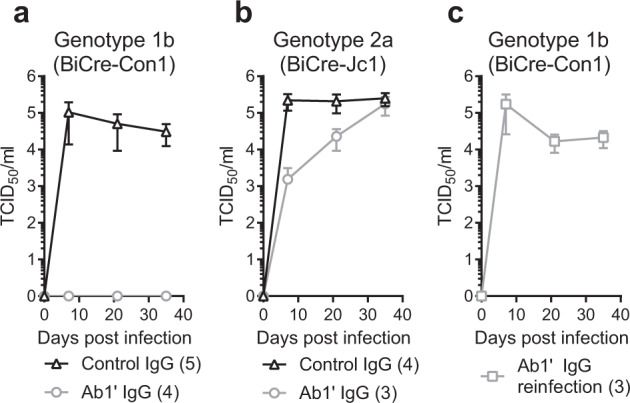
B2.1 A protects against HCV challenge. HCV titers from human liver chimeric mice injected with control IgG or Ab1′ IgG and infected with (**a**) BiCreCon1 (Gt1b), (**b**) BiCreJC1 (Gt2a), (**c**) HCV titers from Ab1′ IgG-treated mice shown in (**a**) following reinfection with BiCreCon1. The number of mice in each group is shown in brackets. Values shown are the mean and SEM.

Our data show compellingly that Ab1′ antibodies raised by immunization with B2.1 A have similar properties to AP33; therefore, we would expect them to neutralize HCV. The serum concentrations of Ab1′ antibodies were not high enough to convincingly neutralize HCVcc, but, when concentrated by affinity-purification, the Ab1′ antibodies efficiently neutralized G451R HCVcc virus (HCV_G451R_) at 2 µg/ml and with an IC_50_ about twice that of AP33 (Supplementary Figures 7a and b, respectively). Encouraged by these preliminary in vitro data, we put all our effort into demonstrating in vivo protection. Rosa26-Fluc mice, which provide a model system for testing HCV entry in vivo, were immunized with B2.1 A Fab and produced a strong E2-specific immune response (Supplementary Fig. 5), but technical difficulties prevented us from challenging the immunized mice with HCVcc-Cre. Instead, a humanized liver chimeric mouse model was used to assess whether Ab1′ antibodies could protect against HCV challenge. Ab1′-containing IgG was purified from sera collected from mice immunized with B2.1A-Fab and control IgG was purified from mock-immunized mice. FNRG mice engrafted with human hepatocytes were dosed with IgG at day −1, 0, and +1 and challenged with HCV on day 0: either with Gt1b virus (BiCre-Con1), or with Gt2a virus (BiCre-JC1). HCV virus titers were determined at day 7, 21, and 35 post-challenge. The animals that were given Ab1′ IgG were completely protected from infection with the Gt1b virus whereas those given control IgG had peak titers of 1 × 10^5^ TCID_50_/ml (Fig. 6a). In a parallel experiment, the Ab1′ IgG gave only partial protection from challenge with Gt2a virus, but even so, there was a 2-log difference in virus titer between the control IgG- and the Ab1′ IgG-treated animals at day 7. Over time, this difference decreased and by day 35 the titers of Gt2a virus were similar (Fig. 6b). In a follow-up experiment, the mice that were protected from the Gt1b HCV infection were re-challenged after the Ab1′ IgG had cleared from their systems. This time all mice succumbed to infection with Gt1b HCV, reaching similar titers to the original control IgG-treated group, thereby confirming that these animals were susceptible to HCV infection (Fig. 6c).

## Discussion

The great variability of viruses such as HIV, influenza, and HCV allows them to evade the immune system in natural infection, and frustrates the development of effective vaccines. Broadly neutralizing antibodies are powerful tools that help to address this problem, because they can be used to identify conserved sites of vulnerability on the virus. In theory, the epitopes of bNAbs provide a basis for the design of vaccines that confer broad protection. In practice, it is highly challenging to make an immunogen based on a conserved epitope that represents only a small portion of a viral protein. More often than not, the epitopes of bNAbs are conformational, i.e. they comprise two or more exposed loops that are adjacent in the folded protein but distant from each other in the linear sequence, so it is difficult to create an antigen that represents the epitope. Even when an epitope is linear, a peptide corresponding to the sequence often does not elicit the desired immune response, probably because it does not adopt the correct conformation and therefore does not adequately mimic the native protein—as in the case of E2_412–423_. To circumvent this problem, we revisited the anti-idiotype network theory^35^. We selected a well-characterized bNAb, AP33, and used it as template to reverse-engineer a surrogate immunogen, B2.1 A, that mimics the antigenic structure of a highly conserved epitope, but is structurally unrelated to the viral protein.

To investigate the extent to which B2.1 A truly mimics the E2 epitope, we obtained a high-resolution structure of the Ab1–Ab2 complex, and compared it to the Ab1–Ag complex^29^. It was interesting to see how the CDR3 of B2.1 A mimics the shape and character of the E2_412–423_ epitope in its β-hairpin conformation without having any sequence similarity. There are in fact very few cases where both the Ab1–Ab2 structure and the Ab1–Ag structure are available at high resolution. A series of papers investigated the structural basis for mimicry of two anti-idiotype antibodies, E225 and E5.2, which bind to a lysozyme-binding antibody, D1.3^50–52^. E225, which binds D1.3 with lower affinity, was found not to be a structural mimic. In contrast, E5.2 and lysozyme, which both bind D1.3 with similar affinities, share 13 contact residues, indicating that E5.2 is a good structural mimic for lysozyme. More recently, Wong and co-workers identified the anti-idiotype Fab E1 to be a partial structural mimic for Dengue virus E protein^53^. These studies support the idea of structural mimicry, which originated purely from the observed binding properties of antibodies^35,36^. Our strategy for characterizing the Ab2s was based on the premise that a structural mimic of an epitope would contact the same residues on the Ab1 as the original antigen. This proved to be true, and the analysis, using a panel of AP33 mutants, resulted in the selection of a bona fide structural mimic.

We demonstrated that B2.1 A was an effective immunogen, completely protecting against HCV Gt1 infection and giving partial protection against HCV Gt2. This result is contrary to the sensitivity to neutralization of these viruses in vitro with AP33. In vitro Gt1b (BiCre-Con1) virus is poorly neutralized by AP33 whereas Gt2a (BiCre-JC1) is much more sensitive (Supplementary Fig. 6) to AP33 neutralization. The protection against Gt2 waned over time; however, it is not clear whether this was due to viral escape or, because protection was not sterilizing, a lack of a functioning immune system in the mice allowed viral load to increase as Ab1′ IgG decreased over time. Notably, reports have shown that Gt2a (Jc1) virus passaged in the presence of AP33 generates resistant mutants whereas Gt1b (Con1) does not, suggesting the sensitivity observed in vivo is more likely due to viral escape^47,54^. This is the first report of an anti-idiotypic approach for HCV; we show that this technique has potential to aid vaccine development for viruses where traditional methods have failed. Selection of both the Ab1 i.e. a bNAb and Ab2, a structural antigen mimic, are used to focus the immune system to recognize important broadly neutralizing epitopes. In order to further develop B2.1 A Fab towards a human vaccine there is potential for further modifications, for example, to humanize the scaffold regions to remove xenotypic epitopes. Another area we are exploring is coupling the Fab to a nanoparticle structure such as ferritin or lumazine synthase which have been used to create multimeric vaccine molecules^55,56^

The E2_412–423_ epitope is conserved in over 90% of all known HCV sequences, but a vaccine that elicits bNAbs to this epitope alone would likely not be sufficient to protect from HCV. It has been shown that when HCV is grown in the presence of AP33, viral escape mutants arise that are resistant to antibody neutralization^47^ although more recent analysis suggests that such mutants have reduced fitness^54^. Therefore, we envisage that an immunogen such as B2.1 A would be used in combination with molecules targeting one or more other conserved B-cell epitopes, to elicit bNAbs with different specificities that could act in concert. Such a structure-based B-cell vaccine would ideally be combined with a T-cell vaccine, in order to elicit cellular immunity as well as protective antibodies.

## Methods

### Cells

Human epithelial kidney cells (HEK)-293T (ATCC CRL-1573), Sp2/0-Ag 14 myeloma cells (ATCC CRL-1581), human hepatoma Huh-7, Huh7.5, and Huh7-J20 were grown in Dulbecco’s modified Eagle’s medium (GIBCO) supplemented with 10% fetal calf serum (FCS), non-essential amino acids (GIBCO), and penicillin/streptomycin. AP33 and B2.1 A hybridoma cells were grown in the same medium supplemented with 10% ultra-low IgG FCS in CELLine CL350 bioreactors (Thermofisher) according to the manufacturer’s instructions. Baby hamster kidney (BHK-21) cells (ATCC CCL-10) were grown in Glasgow minimal essential Eagle’s medium supplemented with 10% newborn calf serum, 4% tryptose phosphate broth, and penicillin/streptomycin. *Spodoptera frugiperda* (Sf9) cells were grown in TC-100 medium (GIBCO) supplemented with penicillin/streptomycin and 10% FCS. Hi Five™ *Trichoplusia ni* cells (BTI-TN-5B1-4) were grown in Express Five™ serum-free medium supplemented with 16 mM L-glutamine. Mammalian cells were incubated at 37 °C in 5% CO_2_, insect cells at 28 °C.

### Antibodies

The anti-E2 murine MAbs AP33 and ALP98, and the rat MAb 3/11 have been described previously^57–59^. The anti-idiotype murine MAb B2.1 A was generated as described herein. MAbs were purified from hybridoma supernatant on HiTrap protein G columns using an Äkta Purifier (GE Healthcare). Fab fragments of AP33 and B2.1 A were made by digesting the respective IgGs for 7 h with immobilized papain, followed by purification using a Nab protein A Plus column (Thermofisher). AP33 Fab was further purified for crystallization by anion exchange on Mono Q 5/50 GL (GE Healthcare) in 20 mM Tris pH 8.5, using a gradient of 0–300 mM NaCl. Biotinylation was carried out using the Immunoprobe^TM^ biotinylation kit (Sigma, BK101). B2.1 A Fab was purified for surface plasmon resonance (SPR) by size exclusion chromatography (SEC) on Superdex 200 GL (GE Healthcare).

### HCV E2_661_

The sequence encoding amino acid residues (aa) 384–661 of HCV genotype 1a strain H77c (GenBank accession no. AF009606), plus an N-terminal 6-histidine tag, was inserted into the pAcGP67-A transfer vector (BD Biosciences). Sf9 cells were co-transfected with the transfer vector and Baculogold™ linearized baculovirus DNA, to make recombinant baculovirus (rBac) expressing the ectodomain of E2 as a secreted, soluble protein. The rBac E2_661_ was cloned by limiting dilution, amplified, and used to infect Hi Five™ cells at a multiplicity of infection (moi) of 1. After 3 days’ incubation the supernatant medium was harvested, filtered through a 0.22 µm membrane, and dialyzed first against 20 mM sodium phosphate pH 6.8, 150 mM NaCl and subsequently against 20 mM sodium phosphate pH 8.0, 300 mM NaCl. Ultrapure imidazole was added to a concentration of 20 mM, and the supernatant loaded onto a HisTrap HP Ni Sepharose column using an Äkta Purifier (GE Healthcare). E2_661_ was eluted with a gradient of 40–200 mM imidazole in 20 mM sodium phosphate pH 8.0, 300 mM NaCl, to separate monomeric molecules from dimers and multimers. Monomeric E2_661_ was purified to homogeneity by size exclusion chromatography (SEC) on a Superdex 200 Increase 10/300 GL column (GE Healthcare).

### Production of Ab2 antibodies

Balb/c mice were immunized with AP33 conjugated to Keyhole Limpet Hemocyanin (KLH). Purified AP33 IgG was conjugated to KLH, in a ratio of 2:1 (wt/wt), using 0.2% glutaraldehyde, and then dialyzed into phosphate-buffered saline (PBS). A primary subcutaneous (s/c) injection of 15 µg AP33-KLH emulsified in Complete Freund’s Adjuvant (CFA) was followed by three s/c injections of 15 µg in Incomplete Freund’s Adjuvant (IFA) at two-weekly intervals. A week after the last injection, serum samples were tested for Ab2 antibodies. The mice received a final intraperitoneal injection of 150 µg AP33-KLH in PBS and five days later their spleen cells were fused with Sp2/0-Ag 14 mouse myeloma cells using 50% PEG. After 12 days of selection in HAT medium, hybridoma colonies were tested for Ab2 production, and serially diluted to obtain single-cell clones. Ab2 antibodies were purified by protein G affinity for further characterization.

### Detection of Ab2 antibodies

Microtitre plates were coated with goat anti-mouse IgG (H + L chains) 40 mg/ml (Jackson Immuno Research) at a dilution of 1:20000 in PBS. Mouse IgG was captured from the hybridoma supernatant during a 1 h incubation at RT. Biotinylated AP33 (b-AP33) was added at 0.1 μg/ml and incubated at RT for 1 h. Bound b-AP33 was detected with streptavidin-conjugated to horse radish peroxidase (HRP) (Sigma S2438, 1:20,000) followed by 3,3′,5,5′-Tetramethylbenzidine (TMB) substrate. Absorbance was measured at 450 nm.

### Competition with AP33

A modified GNA-capture ELISA was used to detect antibodies that compete with AP33 for binding to E2. Soluble genotype 1a E2 was made by infecting BHK cells at 5 PFU/cell with recombinant vaccinia virus expressing aa 384–660 of HCV genotype 1a strain H77c (Owsianka et al 2001). Four days after infection the cells were harvested, washed in PBS and resuspended in lysis buffer (40 mM Tris pH 7.5, 1 mM EDTA, 150 mM NaCl, 1% Igepal CA-630, 20 mM iodoacetamide and complete^TM^ protease inhibitor cocktail (Roche). Nuclei were pelleted by centrifugation at 15,000 × *g* and the cytoplasmic extract containing E2_660_ stored in aliquots at −20 °C. Microtitre plates coated with 2.5 µg/ml *Galanthus nivalis* agglutinin (GNA) were used to capture E2 glycoproteins from cell lysate. Hybridoma supernatant medium or purified Ab2s were mixed with b-AP33, added to the plates and incubated for 1 h at RT. Bound b-AP33 was detected with streptavidin-conjugated to horse radish peroxidase (HRP) (Sigma S2438, 1:20,000) followed by 3,3′,5,5′-Tetramethylbenzidine (TMB) substrate. Absorbance was measured at 450 nm. A decreased signal indicated blocking by Ab2 antibodies of b-AP33–E2 interaction. To test whether Ab2s were specific for AP33, b-AP33 was replaced by b-3/11.

### Binding of Ab3 sera to E2 alanine-scanning mutants

Plasmids encoding alanine substitution mutants of genotype 1a strain H77c E1E2 were constructed as previously described^20^ and used to transfect HEK-293T cells. After two days’ incubation the cells were washed in PBS and cytoplasmic extracts prepared as described above. Microtitre plates coated with 2.5 μg/ml GNA were used to capture E1E2 glycoproteins from cell lysates. MAbs or Ab3 sera were added and incubated for 1 h at RT. Bound antibodies were detected with anti-mouse IgG HRP followed by TMB. Absorbance was measured at 450 nm.

### Binding of Ab2 MAbs to AP33 mutants

Wild type (WT) and mutant AP33 MAbs were produced by transient transfection of HEK cells with expression vectors encoding the appropriate antibody heavy and light chain combinations as previously described^29^. The mutagenesis was carried out on a human-mouse chimera comprising the variable region of AP33 combined with a human constant region, to enable testing of interaction with murine Ab2s. To measure the concentrations of WT and mutant AP33 MAbs, serially diluted IgG-containing media were added, with an appropriate IgG standard alongside, to microtitre plates coated with anti-human IgG antibody (Sigma I-9010; 1:10,000). Captured chimeric MAbs were detected with anti-human IgG HRP (Sigma A-0170; 1:10,000), followed by TMB. Equal amounts of WT and mutant AP33 MAbs were then captured onto microtitre plates coated with anti-human IgG antibody. Ab2 MAbs were added at 1 µg/ml and incubated for 1 h at RT. Bound Ab2 MAbs were detected with anti-mouse IgG HRP (Sigma A4416; 1:5000) followed by TMB. Absorbance was measured at 450 nm.

### Blocking of AP33–B2.1 A interaction by Ab3

Microtitre plates were coated with 1 µg/ml B2.1 A. Dilutions of sera were mixed with b-AP33, added to the plates, and incubated for 1 h at RT. Bound b-AP33 was detected with streptavidin-HRP followed by TMB. Absorbance was measured at 450 nm. A decreased signal indicated blocking of b-AP33–B2.1 A interaction by competing serum antibodies.

### Binding to E2_661_ and peptide inhibition of binding

Microtitre plates were coated with 1 µg/ml of purified E2_661_ (genotype 1a strain H77c). Dilutions of MAbs or sera were added and incubated for 1 h at RT. To test for peptide inhibition, MAbs or sera were pre-incubated with peptides for 30 mins at RT. Bound antibodies were detected with anti-mouse IgG HRP followed by TMB. Absorbance was measured at 450 nm. Titer was defined as the reciprocal of the highest dilution of immune serum that gave a signal at least three times higher than the average signal of pre-immune sera at the same dilution.

### Binding of B2.1 A mutants to AP33

Microtitre plates were coated with 5 µg/ml of AP33 IgG. Serial dilutions of WT and mutant MBP-B2.1 A scFv were added and incubated for 1 h at RT. Binding of MBP-B2.1 A scFv was detected with anti-MBP-HRP (Abcam 49923; 1 µg/ml) followed by TMB. Absorbance was measured at 450 nm.

In all ELISAs, Immulon 2HB microtitre plates were used. The plates were washed three times between each step in PBS plus 0.05% Tween 20 (PBST). PBST was used as a diluent at every step except coating, which was done in PBS. After coating, plates were blocked with 2% skimmed milk in PBST.

### Immunization of mice to generate an Ab3 response

Balb/c or Rosa 26-Fluc mice were immunized with Ab2 IgG, or IgG fragments, conjugated to KLH as above. A primary s/c injection of 50 µg Ab2-KLH emulsified in CFA was followed by up to five s/c booster injections of 50 µg antigen in IFA at two-weekly intervals. In some experiments, the antigen used for alternate booster injections was E2_411–424_ peptide (IQLINTNGSWHINS) conjugated to KLH. Serum samples were taken 7–10 days after the last injection, or after each injection.

To generate Ab1′ IgG for passive transfer into the human chimeric liver mouse model (see below), Rosa 26-Fluc mice were mock-immunized or immunized with 50 μg B2.1 A Fab coupled to KLH. Sera was collected and pooled. The IgG was purified on a HiTrap Protein G column using an ÄKTA™ Pure chromatography system (GE Healthcare). Fractions were pooled and dialyzed into PBS.

### Cloning, expression, and purification of B2.1 A scFv

The genes encoding the variable heavy (vH) and variable light (vL) chain were generated by reverse transcription-polymerase chain reaction from total RNA of the B2.1A-secreting hybridomas cells. The vH and vL segments were linked via a nucleotide sequence encoding a flexible linker (GGSGGSGGGGSGGGGSGGGAS) and this cassette placed downstream from a Igk-leader sequence in a modified mammalian expression vector pDisplay (Life Technologies) to generate a single-chain variable fragment (scFv) expression construct. The vH-linker-vL cassette was subsequently sub-cloned into pLOU3, an *Escherichia coli* expression vector derived from the pMAL-c2x vector (NEB). The construct encodes a maltose-binding protein (MBP)-B2.1 A scFv fusion protein, which carries a 6-histidine sequence at its N-terminus and a tobacco etch virus (TEV) protease cleavage site at the C-terminus of MBP. Briefly, the fusion protein was expressed in *E. coli* strain Rosetta-gami 2(DE3)pLysS (EMD Millipore) and the protein purified from cell extract using HisTrap and MBPTrap columns (GE Healthcare). The protein was then digested with TEV protease and re-applied to the HisTrap column. The unbound fraction containing B2.1 A scFv was further purified on a 16/60 Sephacryl S100 (GE Healthcare) in PBS.

### Crystallization of AP33 Fab complexed with B2.1 A scFv

A Crystal Gryphon Liquid Handling System (Art Robbins Instruments) was used to set up co-crystallization trials using the sitting drop method. B2.1 A scFv was buffer exchanged into crystallization buffer (20 mM Tris–HCl pH 8.5, 80 mM NaCl) and combined with AP33 Fab in a 1:1 molar ratio at a final concentration of 5 mg/mL

### Data collection

Two datasets were obtained from a crystal grown under condition D4 (0.1 M sodium HEPES pH 7.5, 25% (w/v) PEG 8000) of the PEGs suite (Qiagen). One low-resolution set (3 Å) was collected on a Rigaku-MSC Micromax-007 X-ray generator and Saturn 944+ CCD detector at 100 K in-house. The second high-resolution dataset (1.85 Å) was collected from the same crystal at the Diamond Synchrotron facility, IO3 beamline on a PILATUS 3 6 M detector.

### Structure determination and refinement

Molecular replacement using Phaser was used to determine the structure from the low-resolution dataset^60^. Two ensembles were used for the search structure: the AP33 structure (pdb 4gaj) and an in-silico model of B2.1 A created by the SWISS-MODEL server. Three cycles of rigid body refinement and several restrained refinement cycles were performed in Phenix^61^. This low-resolution structure was used to model the high-resolution dataset with the same space group selected with Pointless^62^. Again this model was refined using rigid body refinement in Phenix then alternating cycles of restrained refinement with TLS parameters and water refinement with model inspection and manual refinement on Coot^63^. Contact in the CCP4 suite and the PDBePISA server^64^ were used to analyze inter- and intra-molecular interactions. Finally, MolProbity^65^ was used for model validation which showed that 98% of residues were in favored regions of the Ramachandran plot, with 0.15% in outlier regions, and a Clashscore of 4.86. Pymol was used to generate all images.

### Protein structure accession numbers

The coordinates and structure factors are deposited in the Protein Data Bank under accession code 6SXI.

### Affinity Measurements

SPR experiments were performed on a Biacore X100 instrument at 25 °C. The proteins used were purified to homogeneity by affinity chromatography followed by SEC. AP33 IgG was immobilized on a CM5 chip by standard amine coupling to a level of approximately 400 resonance units (RU). A second flow cell on the same chip was activated and deactivated in the absence of antibody as a negative control. Analytes (E2_661_ or B2.1 A Fab) were injected over the chip at 30 µl/min in buffer (10 mM HEPES pH 7.4, 150 mM NaCl, 3 mM EDTA, 0.05% Tween 20), at concentrations ranging from 0.6 to 80 nM. The sensor surface was regenerated between each cycle with two 60 s injections of 10 mM glycine pH 1.5. Each sensorgram was corrected for nonspecific binding by subtraction of the signal obtained from the negative control flow cell. The sensorgrams were analyzed using Biacore X100 Evaluation software (BIAcore) by applying a 1:1 binding model to obtain kinetic affinity constants.

### Site-directed mutagenesis of B2.1 A ScFv

The BeAtMuSiC program^66^ and the SNP-IN tool (http://korkinlab.org/snpintool/) were used to predict single point mutations that may enhance the affinity of AP33 and B2.1 A. Site-directed mutagenesis was used to generate mutations in the B2.1 A ScFv background. The proteins were expressed in *E.coli* and purified using the His-tag on a BioSprint 15 workstation (Qiagen).

### Hepatitis C virus production and titration

Plasmids encoding the BiCre-Con1 and BiCre-JC1 genomes^67^ were linearized with XbaI then transcribed using T7 RiboMAX^TM^ Express Large Scale RNA Production System (Promega) and purified using RNeasy kit (Qiagen). RNA was electroporated into Huh7.5 cells and infectious virus was harvested every 3 h from 24 h to 96 h post-electroporation then concentrated using a MilliporeSigma™ Amicon™ Bioseparations stirred cells (Merck). Virus titers were determined in a focus-forming unit assay by serial dilution on Huh7.5 cells. Infected cells were stained for the NS5A viral protein as described previously^68^.

### HCVcc neutralization assays

AP33 antibody neutralization assays were performed in the SEAP-reporter cell line Huh7-J20 as described previously^22^. Briefly, cells were seeded at a density of 4 × 10^3^ cells per well into a 96-well plate and incubated at 37 °C overnight prior to infection. Virus was preincubated for 1 h at 37 °C for with AP33 prior to infection at a multiplicity of infection (MOI) of 0.1.

### Engraftment of adult human hepatocytes in FNRG mice

Female FNRG mice^69^ between 8–12 weeks old of age were transplanted with 0.5 × 10^6^ of cryopreserved adult human hepatocytes (Donor 28 from QPS, USA). Hepatocytes were injected by intrasplenic injection during isoflurane anesthesia using an insulin syringe. The peritoneum was sutured using 4.0 VICRYL sutures (Johnson & Johnson, USA) and skin was closed using MikRon Autoclip surgical clips (Becton Dickinson, UK). To facilitate the engraftment of adult human hepatocytes, mice were cycled off and on with the drug nitisinone (Yecuris, USA) according to weight loss and health state. Nitisinone was provided in the drinking water to FNRG mice for maintenance from birth and was retired after surgery. Nitisinone was added again after 10–15% body weight loss was reached. Once the body weight loss was recovered, mice were provided with 10% Nitisinone solution for 2 days before animals were put back on plain drinking water.

### Human albumin ELISA

Human albumin ELISA was used to quantify the level of human hepatocyte engraftment after surgery following a previously published protocol^70^. Mice were bled from the tail vein and serum was serially diluted before use.

### Infection of engrafted FNRG mice after antibody treatment

FNRG mice highly engrafted with human adult hepatocytes according to serum human albumin secretion (at least 1 mg hAlb per mL in peripheral blood) were selected to test the protective capacity of B2.1 A antibodies against BiCreJc1 (genotype 2a) and BicreCon1 (genotype 1b) HCV viruses. Mice were intraperitoneally injected 3 times with 80 μg per mouse of IgG from mice immunized with B2.1 A Fab, or from mock-immunized mice. The first injection was 24 h and the second was 1 h prior HCV infection, the third was 24 h post-HCV infection. Mice were intravenously infected with 10^4^ TCID_50_ of BicreCon1 or BiCreJc1 in PBS. To evaluate the level of infection mice were bled through the tail vein at day 7, 21, and 35 post-infection.

### Animal Ethics

All animal work with infectious agents was conducted in BSL3 facilities in accordance with local rules at Imperial College London, UK. All animal research described in this study was approved by appropriate local Ethics Committee and carried out under United Kingdom Home Office Licenses in accordance with the approved guidelines and under the Animals (Scientific Procedures) Act 1986 (ASPA).

### Reporting Summary

Further information on research design is available in the Nature Research Reporting Summary linked to this article.

## Supplementary information

Supplementary File

Reporting Summary

## Data Availability

The structural data shown in Fig. 2 and Supplementary Figs 1–3 have been deposited in the Protein Data Bank under accession code 6SXI. The data supporting the rest of the findings are available within the main text and supplementary files. The raw data are available from the corresponding author upon reasonable request.
