# Supplementary material for: Development of a structural epitope mimic: an idiotypic approach to HCV vaccine design
Source: NPJ Vaccines. 2021 Jan 8;6:7. doi: 10.1038/s41541-020-00269-1 (PMC7794244; doi:10.1038/s41541-020-00269-1)
Supplement: Supplementary file 1 — Supplementary File [file 41541_2020_269_MOESM1_ESM.pdf]

Supplementary Materials:

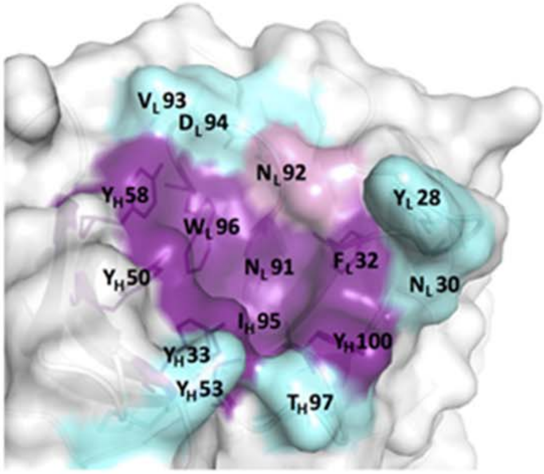

Supplementary Figure 1. The molecular surface of the AP33 antigen-binding pocket. The positions of alanine substitutions that reduced E2 binding by>90% are colored purple. NL92A, which reduced binding by 40%, is in pink. Positions of mutations that had little or no effect on E2 binding are colored cyan.

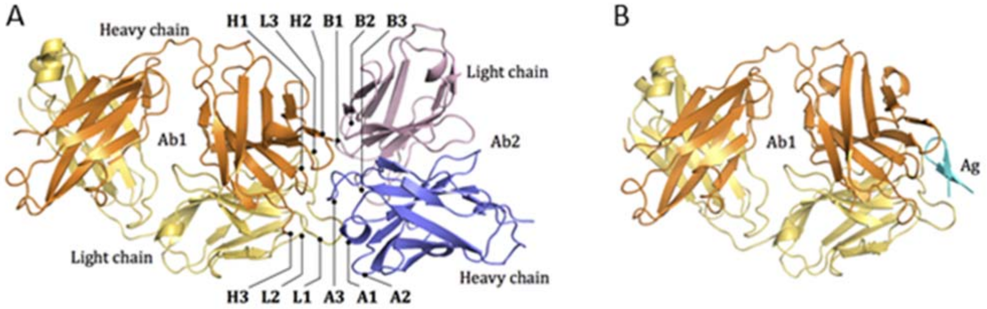

Supplementary Figure 2. B2.1A docks into the AP33 antigen-binding site. Ribbon and surface representation of AP33 Fab (Ab1; heavy chain: orange; light chain: yellow) in complex with (A) B2.1A scFv (Ab2; heavy chain: purple; light chain: pink), and (B) a peptide corresponding to HCV $E2_{412-423}$ (Ag; teal; pdb accession code 4gag).

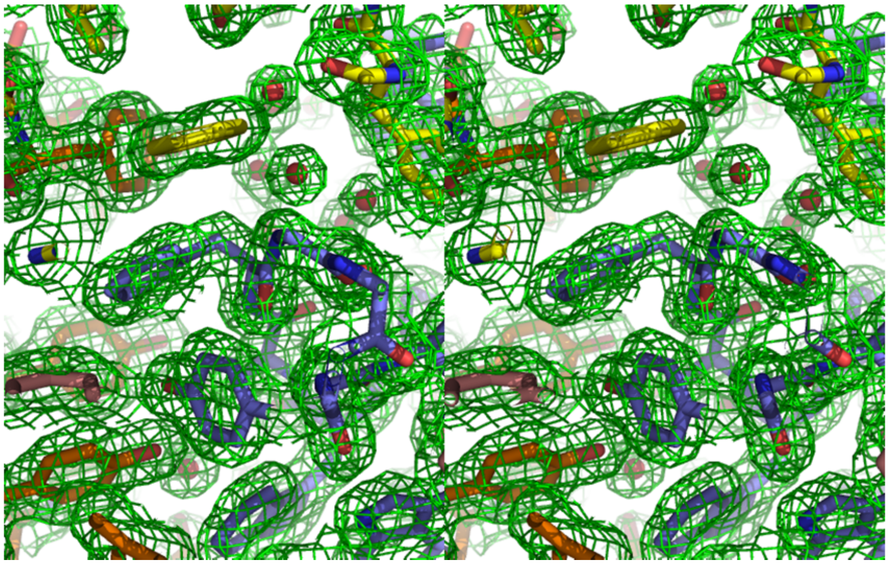

Supplementary Figure 3. Electron density map. A stereo 2Fo-Fc electron density map contoured at 1 sigma around FH98 of B21.A

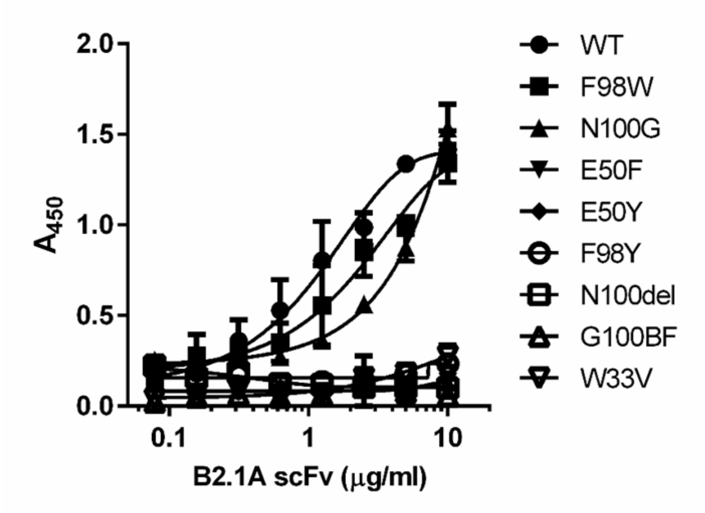

Supplementary Figure 4. Site-directed mutagenesis of B2.1A scFv. Purified B2.1A scFv fusion proteins carrying the indicated mutations were tested by ELISA for binding to immobilized AP33. Each value shows the mean and SD of three experiments. Sigmoidal curves were fitted to the absorbance data.

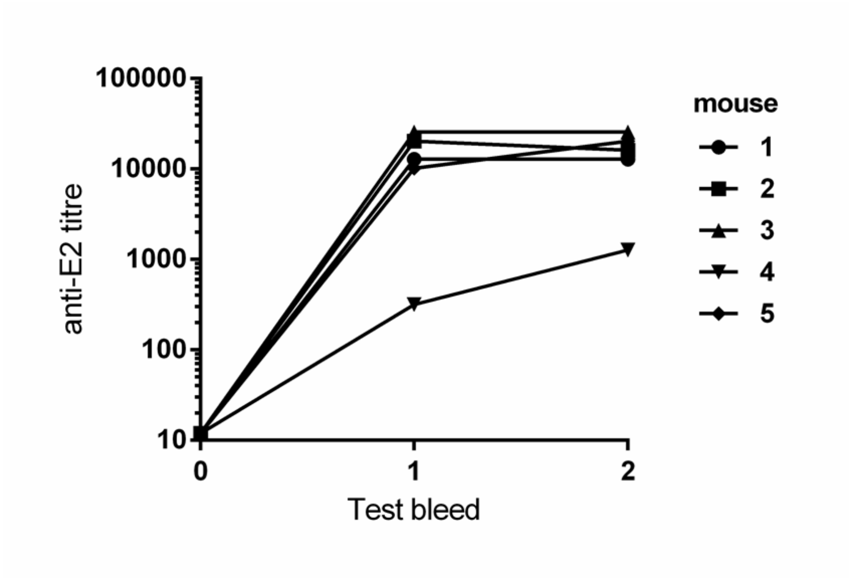

Supplementary Figure 5. Immunogenicity of B2.1A. Rosa26-Fluc mice were given a primary immunization of B2.1A Fab followed by a peptide booster. Test bleeds were taken (0) before immunization, (1) one week after the primary immunization, (2) one week after the booster immunization, and the anti-E2 titre measured by ELISA. Each value represents the geometric mean of three independent titrations.

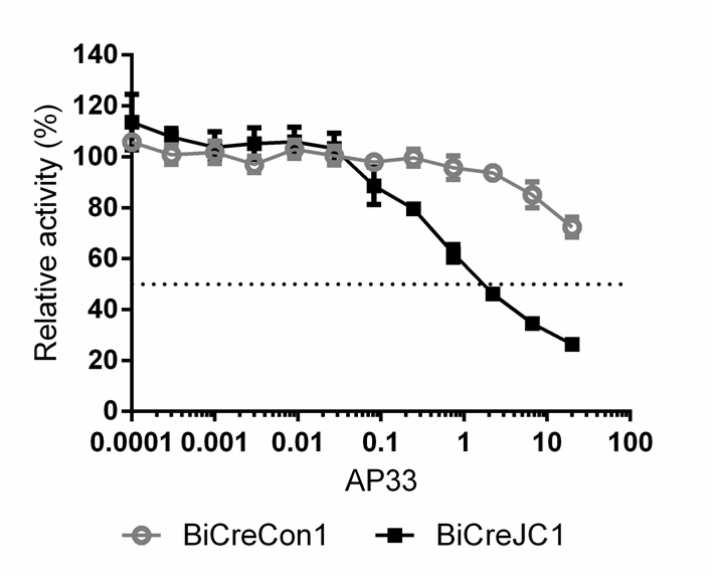

Supplementary Figure 6. AP33 neutralization of virus. The BiCreCon1 (grey, open circles) and BiCreJC1 (black, closed squares) were neutralized by the Ab1 antibody AP33. Each dataset shows the average of four independent experiments and the error bars show the SEM.

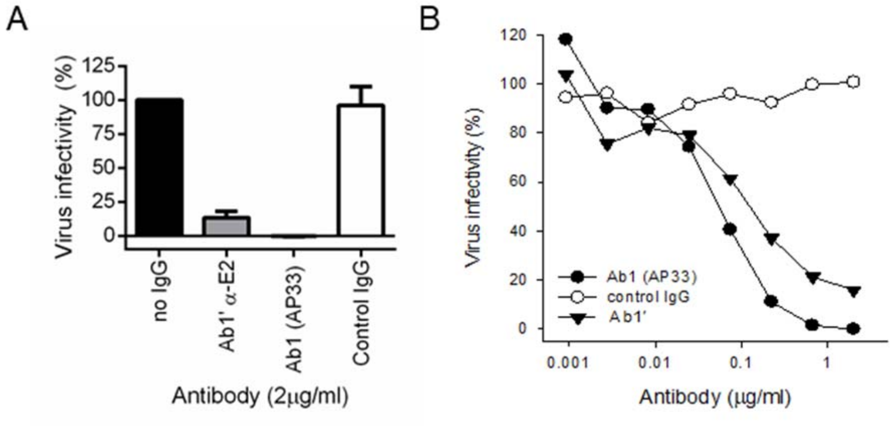

Supplementary Figure 7. B2.1A elicits Ab1′ antibodies that neutralize HCVcc. Immobilized $E2_{661}$ was used to affinity-purify Ab1′ antibodies from the serum of mice immunized with B2.1A. (A) The purified Ab1′ antibodies neutralized genotype 1a HCV_{G451R} virus at 2μg/ml (grey bar). Error bars show the St.dev. This experiment was performed in duplicate. (B) Dose-response of neutralization. The purified Ab1′ antibodies (triangles) neutralized HCV_{G451R} virus with an IC_{50} of 0.15μg/ml. AP33 (closed circles) neutralized the same virus with an IC_{50} of 0.07 μg/ml, while IgG purified from non-immune mouse sera (open circles) had no effect.

Supplementary Table 1. Amino acid conservation of AP33 binding residues

E2 position	Residue[a]	No. of sequences with consensus residue[b]	Variant amino acid residues (No. of sequences)[c]
413	L	30487 (99.84%)	P(19), X(10), F(5), M(4), I(4), V(3), A(2), H(1), Q(1)
415	N	28985 (94.92%)	H(391), R(329), K(318),S(248), Y(155), E(45), D(34), X(20), T(4), Q(4), A(2), C(2), G(1)
418	G	30437 (99.67%)	N(45), D(22), S(16), X(8), R(3), V(2), E(2), A(1)
420	W	30453 (99.73%)	R(38), *(12), C(10), X(7), G(6), L(5), S(2)

[a]The consensus residues represent the majority population in genotypes (gt) 1 to 7.
[b]A total of 30536 E2 sequences containing this region were analysed (3719 gt1, 1361 gt2, 3932 gt3, 789 gt4, 134 gt5, 598 gt6, 3 gt7).
[c]All residues that differ from the consensus are listed, and the number of sequences in which each residue occurs are shown in parentheses. * represents a stop codon and X represents an unknown residue.

Supplementary Table 2. Comparison of the inter-molecular hydrogen bonds in the Ab1-Ag complex and the Ab1-Ab2 complex

Ab1-Ag complex				*Ab1-Ab2 complex*	
**E2 peptide (gag)**	**AP33 binding partner***	**E2 peptide (4gaj)**	**AP33 binding partner***	**B2.1A Fv**	**AP33 binding partner***
Leu-413 Oδ1	Tyr-100 OH (V_H)	Leu-413 Oδ1	Tyr-100 OH (V_H)	Tyr-32 OH (V_H)	**Tyr-33 OH (V_H)**
Asn-415 N	**Tyr-33 OH (V_H)**	Asn-415 N	**Tyr-33 OH (V_H)**	Tyr-32 OH (V_H)	Tyr-53 OH (V_H)
Asn-415 O	**Tyr-50 OH (V_H)**	Asn-415 O	**Tyr-50 OH (V_H)**	Glu-50 Oε1 (V_H)	**Tyr-28 OH (V_L)**
Asn-415 Oδ1	**Tyr-50 OH (V_H)**	Asn-415 Oδ1	**Tyr-50 OH (V_H)**	Asn-100 Nδ2 (V_H)	Asp-27C (V_L)
Gly-418 O	Trp-96 Nε1 (V_L)	Gly-418 O	Trp-96 Nε1 (V_L)	Asn-100 N (V_H)	**Asn-92 O (V_L)**
Val 422 N	**Tyr-28 OH (V_L)**	Gly-418 O	Val-93 N (V_L)	Asn-100 N (V_H)	**Asn-92 Oδ1 (V_L)**
Trp 420 Nε1	Asn-91 O (V_L)	Ser-419 OG	**Asn-92 O (V_L)**	Tyr-100A OH (V_H)	**Tyr-50 OH (V_H)**
Trp 420 N	**Asn-92 Oδ1(V_L)**	Trp 420 Nε1	Asn-91 O (V_L)	Tyr-100A N (V_H)	**Asn-92 O (V_L)**
		Trp 420 N	**Asn-92 Oδ1 (V_L)**	Arg-30 Nε (V_L)	Glu-27 Oε2 (V_L)
				Thr-31 Oγ1 (V_L)	Asp-94 Oδ2 (V_L)
				Tyr-49 OH (V_L)	Thr-57 O (V_H)
				Leu-50 O (V_L)	Arg-64 NH1 (V_H)
				Ser-52 Oγ (V_L)	Arg-64 NH1 (V_H)
				Tyr-96 OH (V_L)	**Tyr-28 OH (V_L)**

AP33 residues that form hydrogen bonds in both the Ab1-Ag complex and Ab1-Ab2 complex are shown in bold typeface; *AP33 residues that when mutated to alanine gave a reduction in binding greater than 90% are underlined (Supplementary Table 1).

Supplementary Table 3. Data collection and refinement statistics (molecular replacement)

**Data collection**	**AP33:B2.1A**
Space group	P 21 21 21
**Cell dimensions**	
a, *b*, *c* (Å)	41.09, 93.28, 177.04
a, b, g (°)	90, 90, 90
Resolution (Å)	64.21-1.85 (1.92-1.85) *
R_{merge} (%)	5.7 (5.5)
Average *I* / s*I*	15.25 (2.46)
Completeness (%)	92.0 (94.4)
Redundancy	4.0 (3.9)
**Refinement**	
Resolution (Å)	64.2 – 1.85 (1.92 – 1.85)
No. reflections	55,005 (5574)
R_{work} / R_{free}	0.16 / 0.21 (0.20 / 0.26)
No. atoms	5639
Protein	5127
Ligand/ion	6
Water	506
***B*-factors**	
Protein	26.2
Ligand/ion	39.6
Water	34.5
**R.m.s. deviations**	
Bond lengths (Å)	0.007
Bond angles (°)	1.13

*One crystal for the structure. *Values in parentheses are for highest-resolution shell.

Supplementary Table 4. B2.1A scFv mutations with predicted effects on AP33-B2.1A binding

Chain	Mutation	Selection	Notes
V_H	Trp-33 to Val	Structure	Reduce steric hindrance of Trp-33 on AP33 V_LTyr-28
V_H	Glu-50 to Phe	Prediction	Increased binding affinity (ΔΔG=-1.28 kcal/mol)*
V_H	Glu-50 to Tyr	Prediction	Increased binding affinity (ΔΔG=-1.24 kcal/mol)*
V_H	Phe-98 to Tyr	Structure	Increase antigen mimicry (around Trp-420)
V_H	Phe-98 to Trp	Structure	Increase antigen mimicry (around Trp-420)
V_H	Asn-100 to Gly	Structure	Reduce steric hindrance of Asn-100 on AP33 V_LAsn-92
V_H	Asn-100 deletion	Structure	Reduce steric hindrance of Asn-100 on AP33 V_LAsn-92
V_H	Gly-100B to Phe	Prediction	Increased binding affinity (ΔΔG=-0.61 kcal/mol)*

*ΔΔG = change in binding entropy as predicted by BeAtMuSiC
