# Supplementary material for: Development of a structural epitope mimic: an idiotypic approach to HCV vaccine design
Source: NPJ Vaccines. 2021 Jan 8;6:7. doi: 10.1038/s41541-020-00269-1 (PMC7794244; doi:10.1038/s41541-020-00269-1)
Supplement: Supplementary file 2 — Reporting Summary [file 41541_2020_269_MOESM2_ESM.pdf]

nature research

Corresponding author(s): Arvind H. Patel

Last updated by author(s): 4 December 2020

Reporting Summary

Nature Research wishes to improve the reproducibility of the work that we publish. This form provides structure for consistency and transparency in reporting. For further information on Nature Research policies, see our Editorial Policies and the Editorial Policy Checklist.

Statistics

For all statistical analyses, confirm that the following items are present in the figure legend, table legend, main text, or Methods section.

n/a	Confirmed	
☐	☒	The exact sample size (n) for each experimental group/condition, given as a discrete number and unit of measurement
☒	☐	A statement on whether measurements were taken from distinct samples or whether the same sample was measured repeatedly
☐	☒	The statistical test(s) used AND whether they are one- or two-sided<br>*Only common tests should be described solely by name; describe more complex techniques in the Methods section.*
☒	☐	A description of all covariates tested
☐	☒	A description of any assumptions or corrections, such as tests of normality and adjustment for multiple comparisons
☐	☒	A full description of the statistical parameters including central tendency (e.g. means) or other basic estimates (e.g. regression coefficient) AND variation (e.g. standard deviation) or associated estimates of uncertainty (e.g. confidence intervals)
☐	☒	For null hypothesis testing, the test statistic (e.g. F, t, r) with confidence intervals, effect sizes, degrees of freedom and P value noted<br>*Give P values as exact values whenever suitable.*
☒	☐	For Bayesian analysis, information on the choice of priors and Markov chain Monte Carlo settings
☒	☐	For hierarchical and complex designs, identification of the appropriate level for tests and full reporting of outcomes
☒	☐	Estimates of effect sizes (e.g. Cohen's d, Pearson's r), indicating how they were calculated

Our web collection on statistics for biologists contains articles on many of the points above.

Software and code

Policy information about availability of computer code

Data collection	Protein purification, affinity measurement and X-ray crystallography data were collected as described in the manuscript. ELISA data were collected in Excel
Data analysis	GraphPad Prism 6.07 was used for data analysis

For manuscripts utilizing custom algorithms or software that are central to the research but not yet described in published literature, software must be made available to editors and reviewers. We strongly encourage code deposition in a community repository (e.g. GitHub). See the Nature Research guidelines for submitting code & software for further information.

Data

Policy information about availability of data

All manuscripts must include a data availability statement. This statement should provide the following information, where applicable:

- Accession codes, unique identifiers, or web links for publicly available datasets
- A list of figures that have associated raw data
- A description of any restrictions on data availability

The structural data shown in Fig.2 and Supplementary Figs 1-3 has been deposited in the Protein Data Bank under accession code 6XSI. The data supporting the rest of the findings are available within the main text and supplementary files. The raw data are available from the corresponding author upon reasonable request.

Field-specific reporting

Please select the one below that is the best fit for your research. If you are not sure, read the appropriate sections before making your selection.

☒ Life sciences ☐ Behavioural & social sciences ☐ Ecological, evolutionary & environmental sciences

For a reference copy of the document with all sections, see nature.com/documents/nr-reporting-summary-flat.pdf

Life sciences study design

All studies must disclose on these points even when the disclosure is negative.

Sample size	For immunization experiments we used a group size of six animals that was determined based on the resource equation
Data exclusions	No data were excluded
Replication	Replicates of experiments were performed
Randomization	No randomization performed
Blinding	N/A

Reporting for specific materials, systems and methods

We require information from authors about some types of materials, experimental systems and methods used in many studies. Here, indicate whether each material, system or method listed is relevant to your study. If you are not sure if a list item applies to your research, read the appropriate section before selecting a response.

Materials & experimental systems

n/a	Involved in the study	
☐	☒	Antibodies
☐	☒	Eukaryotic cell lines
☒	☐	Palaeontology and archaeology
☐	☒	Animals and other organisms
☒	☐	Human research participants
☒	☐	Clinical data
☒	☐	Dual use research of concern

Methods

n/a	Involved in the study	
☒	☐	ChIP-seq
☒	☐	Flow cytometry
☒	☐	MRI-based neuroimaging

Antibodies

Antibodies used	Anti-mouse IgG HRP was purchased from Sigma (A4416). The anti-E2 murine MAbs AP33 and ALP98, and the rat MAb 3/11 have been described previously (references 56-58). The anti-idiotype murine MAb B2.1A was generated as described herein.
Validation	See references 56-58 (and numerous other published papers) for details of MAb AP33, ALP98, and 3/11. Details of MAb B2.1A are in the present manuscript.

Eukaryotic cell lines

Policy information about cell lines

Cell line source(s)	Human epithelial kidney cells (HEK)-293T (ATCC CRL-1573); Sp2/0-Ag 14 myeloma cells (ATCC CRL-1581); Baby hamster kidney (BHK-21) cells (ATCC CCL-10); Hi Five™ Trichoplusia ni cells (BTI-TN-5B1-4) Thermo Fisher Scientific. Huh7 cells were obtained from J Dubuisson and has been described in numerous publications. Huh7-J20 are derivatives of Huh7 cells that were described in our paper Iro et al., Antiviral Research 83 (2009) 148–155.
Authentication	None of the cell lines described were authenticated
Mycoplasma contamination	All cell lines were negative for mycoplasma contamination.
Commonly misidentified lines (See ICLAC register)	N/A

Animals and other organisms

Policy information about studies involving animals; ARRIVE guidelines recommended for reporting animal research

Laboratory animals	Balb-C mice male and female, 6-8 weeks old; Rosa26-FLuc mice, male and female, 6-8 weeks old; Female FNRG mice (ref 68) between 8-12 weeks old
Wild animals	N/A
Field-collected samples	N/A
Ethics oversight	All animal research described in this study was approved by appropriate local Ethics Committees and carried out under a United Kingdom Home Office Licenses in accordance with the approved guidelines and under the Animals (Scientific Procedures) Act 1986 (ASPA).

Note that full information on the approval of the study protocol must also be provided in the manuscript.
